# Evaluation of the Clinitek®, a point-of-care urinalysis system for the measurement of clinically significant urinary metabolites and detection of haematuria in *Schistosoma haematobium* infected children in southern Côte d’Ivoire

**DOI:** 10.1186/s13071-019-3555-z

**Published:** 2019-06-13

**Authors:** Gordana Panic, Beatrice Barda, Jana Kovač, Jean T. Coulibaly, Jennifer Keiser

**Affiliations:** 1 Department of Medical Parasitology and Infection Biology, Swiss Tropical and Public Health Institute, Basel, Switzerland; 2 University of Basel, Basel, Switzerland; 3 Present Address: Department of Surgery and Cancer, Division of Systems and Digestive Medicine, Imperial College London, London, UK; 4 Unité de Formation et de Recherche Biosciences, Université Félix Houphouët-Boigny, Abidjan, Côte d’Ivoire; 5 Centre Suisse de Recherches Scientifiques en Côte d’Ivoire, Abidjan, Côte d’Ivoire

**Keywords:** Electronic analyser, Haematuria, *Schistosoma haematobium*, Urinalysis, Urinary dip-stick

## Abstract

**Background:**

Urinary schistosomiasis, caused by *Schistosoma haematobium*, remains a significant public health problem worldwide, despite years of efforts to control it. Haematuria is one of the notable indirect indicators of *S. haematobium* infection and is commonly assessed along with other routine screens using a urinary dipstick test. A portable “field friendly” electronic analyser would offer an automated and thus more objective read-out compared to visual-read dipstick methods.

**Methods:**

Within the framework of a Phase 2 praziquantel dose finding study in preschool- and school-aged children infected with *S. haematobium*, in southern Côte d’Ivoire, we compared a visual-read of the urine dipstick strips (Multistix PRO, Siemens Healthcare Diagnostics) to an automated reader (CLINITEK Status+ analyser™ Siemens Healthcare Diagnostics). Urine samples were collected from 148 pre-school aged and 152 school-aged children for urinalysis. Values were compared using a linear weighted kappa statistic and Bland–Altman analysis.

**Results:**

A very good correlation between the two methods for nitrites and haematuria was observed (κ coefficient of 0.88 and 0.82, respectively), while a good correlation was observed for leukocytes (κ coefficient of 0.63) A moderate to fair correlation was calculated (κ coefficient ≤ 0.6) for all other parameters. When the results were stratified according to infection intensity, the agreements were stronger from the high infection intensity sample measurements, for most of the parameters.

**Conclusion:**

Our results demonstrate the device’s utility in detecting haematuria and nitrites but underline the need for further development of this tool in order to improve its performance in the field.

## Background

*Schistosoma haematobium* is one of the three main human species of schistosomes and causes the urinary form of schistosomiasis [1–3]. The flukes reside in blood vessels surrounding the urinary tract and the eggs they produce become trapped in the tissue, causing inflammation and leading to ulceration and pathology of the bladder and kidney. Consequently, urinalysis is recognised as a rapid indirect tool for assessment of urinary tract morbidity caused by schistosomiasis [4, 5]. Haematuria is defined as the presence of red blood cells in urine. It can be detected by macroscopic urinalysis, where the presence of blood in urine is seen by naked eye, or *via* microscopic urinalysis, where the use of a microscope is needed [6]. Studies have shown that people shedding *S. haematobium* eggs also commonly have haematuria [1, 7–11]. Therefore, although epidemiological surveys and studies use egg counts directly, haematuria is one of the first indicators of urinary schistosomiasis in general clinical evaluations and can also indicate morbidity caused by the disease [12, 13].

There are several ways to detect haematuria, depending on the resources, conditions and environment. Commercial dipstick tests designed to detect haem in urine have proven to be a good proxy for detecting and mapping urinary schistosomiasis [12]. Their simplicity and rapid turnover are of particular importance when it comes to low-resource settings and where intervention is urgently required. In addition to blood in urine, other parameters are also detected and quantified using the urine stick: leukocyturia (LEU); nitrites (NIT); urobilinogen (UBG); proteinuria (PROT); pH, specific gravity (SG); ketones (KET); bilirubin (BIL); and glucose (GLU) (Table 1). These parameters are commonly used to diagnose different diseases and some parameters, such as proteinuria, can be indicators of schistosomiasis-related morbidity [14–16]. All the parameters can be read out visually; however, the consistency of the read-outs may vary between individuals. In clinical settings, an automatic urinalysis reader is typically used as it eliminates bias due to subjective reading by naked eye and increases the throughput of read-outs (each read only requires one minute to process) [17]. Portable, battery-operated versions of these devices have been introduced which would, in theory, render them usable in more remote areas [6, 18].

**Table 1:** Number and (%) of manual and analytical evaluation of various parameters

Parameter	Descriptive	Numeric	Rank	Visual read (out of *N* = 300)	CLINITEK (out of *N* = 300)
Leukocytes (Leu/µl)	neg/normal	0	0	139 (46.3)	102 (34.0)
	trace	15	1	38 (12.7)	73 (24.3)
	+	70	2	39 (13.0)	73 (24.3)
	++	125	3	68 (22.7)	25 (8.3)
	+++	500	4	14 (4.7)	23 (7.7)
			nd	2 (0.7)	4 (1.3)
Nitrites^a,b^	neg/normal	na	0	272 (90.7)	263 (87.7)
	positive	na	2	28 (9.3)	35 (11.7)
			nd	0 (0)	2 (0.7)
Urobilinogen^a^ (E.U./dl; mmol/l)	neg/normal	0.2,1; 3.2, 16	0	292 (97.3)	280 (93.3)
	+	2; 33	2	5 (1.7)	9 (3.0)
	++	4; 66	3	2 (0.7)	6 (2.0)
	+++	≥ 131; 8	4	1 (0.3)	2 (0.7)
			nd	0 (0)	3 (1.0)
Proteins (g/l; mg/dl)	neg/normal	0	0	154 (51.3)	96 (32.0)
	trace	0.2; trace	1	59 (19.7)	31 (10.3)
	+	0.3; 30	2	50 (16.7)	69 (23.0)
	++	1; 100	3	29 (9.7)	76 (25.3)
	+++	≥ 3; 300	4	8 (2.7)	26 (8.7)
			nd	0 (0)	2 (0.7)
Blood (Eur/µl)	neg/normal	0	0	107 (35.7)	111 (37.0)
	trace	10	1	38 (12.7)	40 (13.3)
	+	25	2	14 (4.7)	18 (6.0)
	++	80	3	45 (15.0)	51 (17.0)
	+++	200	4	95 (31.7)	80 (26.7)
			nd	1 (0.3)	0 (0)
Ketone bodies (mg/dl)	neg/normal	0	0	278 (92.7)	264 (88.0)
	trace	5	1	20 (6.7)	33 (11.0)
	+	15	2	1 (0.3)	2 (0.7)
	++	40	3	0 (0)	0 (0)
	+++	80	4	0 (0)	0 (0)
	++++	160	5	0 (0)	0 (0)
			nd	1 (0.3)	0 (0)
Bilirubin^a^	neg/normal	na	0	220 (73.3)	220 (73.3)
	+	na	2	51 (17.0)	74 (24.7)
	++	na	3	25 (8.3)	5 (1.7)
	+++	na	4	3 (1.0)	1 (0.3)
			nd	1 (0.3)	0 (0)
Glucose^c^ (g/dl; mg/dl)	neg	0	0	299 (99.7)	297 (99.0)
	+	0.25; 250	2	0 (0)	3 (1.0)
			nd	1 (0.3)	0 (0)

*Key*: na, not applicable; nd, no data; +, small; ++, moderate; +++, large; ++++, very large

^a^One or both read-outs don’t offer a trace (rank 1) read, only negative or gradients of positive

^b^Dipsticks offer gradients of positive readout (+, ++, +++) but machine reads only negative or positive

^c^Full glucose read-out gradients not shown as almost all children were negative except for 3 small positives

In this study, we compared the performance of the CLINITEK Status+ Analyser™ (Siemens Healthcare Diagnostics, Erlangen, Germany), to manual read-outs of urine strips (Multistix PRO, Siemens Healthcare Diagnostics).

## Methods

Urine samples were collected as part of a Phase 2 dose-finding clinical trial with praziquantel in *S. haematobium*-infected children [19]. Collectively, 174 school-aged and 170 preschool-aged children were enrolled in the trial, which took place in November 2015 in Côte d’Ivoire, in the health district of Adzopé.

Urine samples were collected prior to treatment as a part of physical examination. The urine dipsticks were first read by a trained member of medical staff and, immediately after the visual analysis, with the CLINITEK Status+ Analyser, according to manufacturer’s instructions. Results were recorded on the case report forms (CRF) of each patient and transferred into electronic form (Microsoft Excel, 2011) at the end of the trial. For the leukocyte, urobilinogen, proteins, blood, ketone bodies and bilirubin parameters, findings were compared employing linear weighted Cohen’s kappa (κ) coefficient statistic using MATLAB 2018a (Mathworks Inc., Natick, USA) [20] to determine the agreement in measured values between both methods for each parameter. An unweighted Cohen’s kappa statistic was used for the nitrites parameter, as it only provides positive/negative assessments. The agreement between the two used techniques was calculated by the Cohen coefficient κ using the following grading: very good (0.81–1.00); good (0.61–0.80); moderate (0.41–0.60); fair (0.21–0.40); and poor (≤ 0.2). For the continuous metrics, the pH and specific gravity (SG), a Bland–Altman plot, created in Excel was used to visualise agreement. Results were then also stratified according to infection intensity using WHO standards for *S. haematobium* infection intensity classification: egg count of 1–49 eggs per 10 ml urine was regarded as light; and egg count of ≥ 50 eggs/10 ml urine was regarded as heavy infection [21].

## Results

152 school- and 148 preschool-children provided urine samples where both measurements with the CLINITEK Status+ Analyser and by visual readout could be obtained and were included in our comparison (Table 1). Over half the participants were female (54.7%). The mean age, weight and height of the study population was 6.4 ± 0.2 years, 19.9 ± 0.4 kg and 112.2 ± 1.1 cm, respectively. Their average temperature was 36.6 ± 0.18 °C and their mean haemoglobin measurements were 11.0 ± 0.1 g/dl. The children harboured predominantly light intensity infections (83.7% of samples) and this was true for both pre-school-aged (90.5% of samples) and school-aged children (72.4% of samples). The mean egg count for light intensity infections was 7.2 ± 1.0 eggs/10 ml urine, whereas those harbouring heavy infections averaged 97.9 ± 48.8 eggs/10 ml urine. With respect to relevant co-infections, 10.7% of the children also had an *S. mansoni* infection, none presented with other helminth infections and 42.3% tested positive for malaria using rapid diagnostic tests, though none of these presented with clinically significant symptoms.

Of the children found to have eggs in their urine, haematuria was detected in 64.3% of their samples by visual read-out and in 63.0% of their samples by machine read-out (Table 1). When stratified according to infection intensity, blood in urine was predictive of light infection in 58.1% and 56.9% of the children using the manual and machine read-out, respectively, and of heavy infection in 92.3% of the children, regardless of the method used.

The agreement between the two methods for each parameter is presented in Table 2. The observed agreement between the two techniques was fair for bilirubin and ketone bodies (κ = 0.38 and 0.32, respectively), moderate for urobilinogen and proteins (κ = 0.60 and 0.44, respectively), good for leukocytes (κ = 0.63) and very good for nitrites and haematuria (κ = 0.88 and κ = 0.82, respectively). Only 3 children had glucose in urine according to the automatic readout whereas none was detected using the manual readout, hence a kappa statistic could not be calculated for this parameter. When stratified according to infection intensity, the agreements are stronger in heavy infection samples for all parameters, except for leukocytes, where agreement was inferior, and for nitrites where the agreement statistic was not calculable.

**Table 2:** Agreement between analytical vs. manual evaluation of various parameters on the Multistix PRO urine dipstick tests

Parameter	Light infection			Heavy infection			Combined		
	kappa	SE	*Z*	kappa	SE	*Z*	kappa	SE	*Z*
Leukocytes (LEU)	0.64	0.06	12.02	0.49^d^	0.15	3.74	0.63	0.06	13.33
Nitrites (NIT)^a^	0.87	0.05	13.43	nc	nc	nc	0.88	0.05	14.78
Urobilinogen (URO)	0.57	0.20	6.83	0.74	0.32	4.52	0.60	0.18	8.66
Proteins (PROT)	0.38	0.07	4.64	0.48^e^	0.14	2.92	0.44	0.06	6.25
Blood (BLO)	0.79	0.04	15.64	0.81	0.12	7.35	0.82	0.03	17.59
BLO yes/no only^b^	0.81	0.04	12.71	1.00	0.00	7.21	0.83	0.03	14.41
Ketone bodies (KET)	0.30^c^	0.18	2.77	0.39^f^	0.28	1.74	0.32	0.15	4.65
Bilirubin (BIL)	0.32	0.12	5.08	0.61	0.19	5.28	0.38	0.10	7.15
Glucose (GLU)	nc	nc	nc	nc	nc	nc	nc	nc	nc

*Note*: *P*-values for all parameters measured were lower than 0.0001 except where noted

^a^Unweighted kappa statistic was applied, as the parameter is binary

^b^Collapsed amount of blood measure to just positive or negative for haematuria

^c^*P* = 0.0056

^d^*P* = 0.0002

^e^*P* = 0.0035

^f^*P* = 0.0825

*Abbreviation*: nc, not calculable, due to lack of values in one measuring technique

**Fig. 1:**
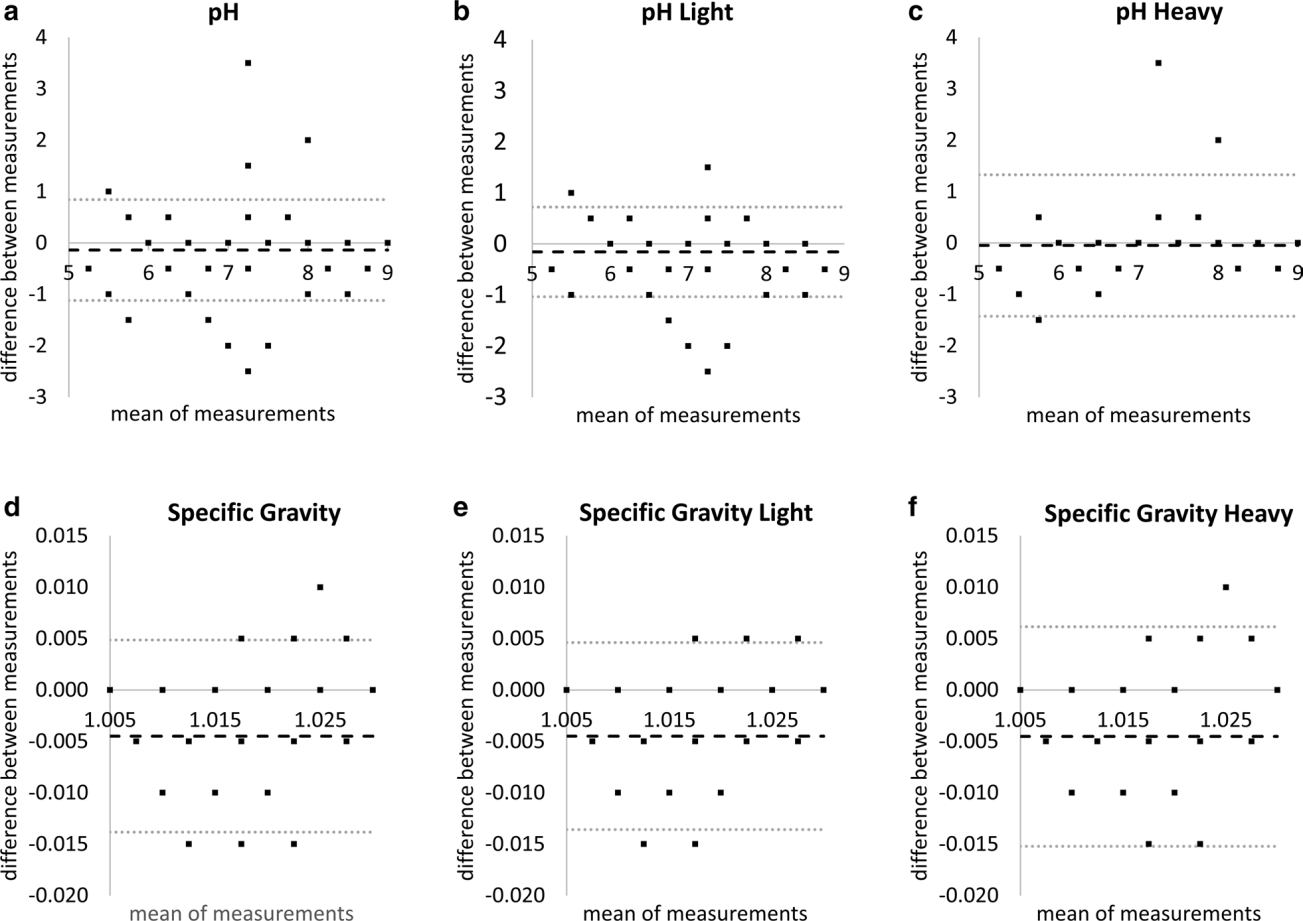
Difference in measurement between a visual *vs* a CLINITEK readout for pH and specific gravity. Bland–Altman plots are shown to indicate difference in agreement for **a** pH for all samples as well as pH stratified by: **b** light infection intensity and **c** high infection intensity. The parallel plots are shown for specific gravity (**d**–**f**). Dashed black line indicates the mean difference while the grey dotted lines are the upper and lower limits of agreement

The mean pH was 7.31 and 7.44 for the visual and CLINITEK read-outs, respectively, while the mean SG values were 1.013 and 1.018, respectively. There was a moderate agreement between the two read-out methods with respect to these two parameters (Fig. 1). The Bland–Altman non-parametric analysis revealed a mean bias of − 0.14 (95% CI: − 0.20 to − 0.08) for pH, with an underestimation by machine readout influenced largely at pH values of 6.5 to 7.5. The mean bias for SG was − 0.004 (95% CI: − 0.005 to − 0.004) wherein underestimation by machine read-out occurred between values of 1.010 and 1.025. The Pearson’s correlation coefficient between the two read-outs was 0.88 for pH and 0.72 for SG. These metrics, stratified according to infection intensity, are presented in Table 3.

**Table 3:** Comparison and agreement of pH and specific gravity measures between the visual vs machine read-out, stratified by infection intensity

Parameter	Infection intensity	Mean ± SD from visual read	Mean ± SD from machine read	Mean bias (95% CI)	Pearson’s *r*
pH	Combined	7.31 ± 0.97	7.44 ± 1.03	− 0.14 (− 0.20, − 0.08)	0.88
	Light infection	7.37 ± 0.91	7.53 ± 1.00	− 0.16 (− 0.21, − 0.10)	0.89
	Heavy infection	6.94 ± 1.16	6.99 ± 1.05	− 0.05 (− 0.24, 0.15)	0.80
Specific gravity	Combined	1.013 ± 0.007	1.018 ± 0.005	− 0.004 (− 0.005, − 0.004)	0.72
	Light infection	1.013 ± 0.007	1.017 ± 0.005	− 0.004 (− 0.005, − 0.004)	0.71
	Heavy infection	1.015 ± 0.007	1.020 ± 0.006	− 0.005 (− 0.006, − 0.003)	0.70

## Discussion

The use of an automated urine dipstick read-out machine would help in diagnosing haematuria and key clinical parameters with high accuracy, increased speed compared to manual read-out and reduced inter-reader variability. Point-of-care testing requiring little equipment and minimal training can improve healthcare globally, in particular in settings where high quality medical care is a challenge [22]. Many studies have compared performance of visual read-out *vs* automated read outs (using CLINITEK or other devices), where they tended to demonstrate on-par or superior performance of the analyser as compared to visual read-out [17, 23]. However, they were largely evaluated in controlled laboratory or hospital settings [24, 25], and usually focused on a single parameter to about three parameters [15, 26]. As such, limited data are available to support the use of automated dipstick readers in the field. We compared, for the first time, visual readout to the CLINITEK system in children infected with *S. haematobium* in a rural African setting. We found that the visual read-out and the CLINITEK device had very good agreement for the measurement of haematuria (κ = 0.83) as well as for nitrites (κ = 0.88), a biomarker for urinary tract infections [27]. It also had good agreement for leukocytes, which somewhat reflects a previous finding in routine hospital tests, where agreement between visual and automated read-outs (using another analyser) was very good for nitrites and good for haematuria albeit poor for leukocytes [17]. The agreement was, however, only fair to moderate for all other biomarkers. For example, the machine read-out provided on average lower readings for pH and SG than the visual read-out. In one study, the Multistix combined with the CLINITEK was found to over- and underestimate urine pH for high and low pH values, respectively [16]; however, in our study the disagreements were stronger for the middle pH ranges. Moreover, as the specific gravity measurements are affected by pH, the CLINITEK automatically adds 0.005 to the specific gravity read-out for pH values ≥ 6.5, but this should have resulted in a slight overestimation when comparing the two.

It is not altogether clear why the agreement for the other parameters would be underwhelming, but a few factors may have influenced some of the poorer outcomes. First, although the machine is portable and does not require much power, it still appears to be more suitable for a laboratory setting than the humid tropical heat of our sites. Specifically, we were not able to keep the machine in sunlight as this caused it to sporadically shut down, requiring a restart. Also, it is advised to store the reagent strips in a closed dry container in a cool place and not expose them to temperatures > 30 °C and, although we kept the container closed throughout the day, it is not possible to avoid the other conditions during a field trial. Secondly, instructions from Siemens also warned that high values of some biomarkers would affect the readout values of other ones: for example, a high urine pH (pH > 8) would falsely increase proteinuria read-outs, and indeed about 24–32% of our samples had a pH > 8. Finally, a limitation of this study is that, with exception to measuring the suitability of haematuria read-outs as a proxy for *S. haematobium* infection, it was not designed to discern which read-out method, the visual or automated, better agrees with gold standard laboratory assays for the different parameters, only how much they agree.

The detection of eggs in urine correlated with detection of haematuria only in 63% and 64% of the cases, for machine and visual read-out, respectively. This is in contrast to a meta-analysis on the performance of urine dipstick tests in detection of urinary schistosomiasis, where dipstick sensitivity and specificity for detection of egg-positive urine were 81% and 89%, respectively [12]. The authors did note that sensitivity was highest among heavy infection intensities and dropped to only 65% for individuals with low-intensity infections, and our study mirrors this trend. Interestingly, in high intensity infections, the agreement between haematuria detection between the two methods was 1:1. Indeed, the agreement between most parameters improved when high intensity samples were measured, which hints that they might be clinically relevant for schistosomiasis-related morbidity. Our findings reaffirm that, although haematuria might be a good indicator of morbidity due to urinary schistosomiasis, its utility as a diagnostic tool in areas of low-intensity infections is dubious, therefore underscoring the urgent need for more sensitive point-of-care diagnostic tools [28].

## Conclusions

The agreement between visual and automated readout of the Multistix Pro urine dipsticks was fair to moderate for most parameters. However, a very good agreement was observed for haematuria and nitrites, two parameters in detecting *S. haematobium* as well as urinary tract infections, which are often associated with schistosomiasis. We believe that the rural tropical environment does affect the performance of both the sticks and the analyser, which would affect their utility for measuring many of the parameters in such a setting. The study thus demonstrates that the machine is currently not superior to visual read-outs in field settings and offers insight into possible future improvements for its use in field studies.

## Data Availability

Data supporting the conclusions of this article are included within the article. The datasets used and/or analysed during the current study are available from the corresponding author upon reasonable request.

## Abbreviations

LEU: Leukocytes
NIT: Nitrites
URO: Urobilinogen
PROT: Proteins
BLO: Blood
KET: Ketone bodies
BIL: Bilirubin
GLU: Glucose
κ: kappa statistic
